# Are we failing to teach cardiopulmonary resuscitation (CPR) in schools? A pilot study to assess CPR and automated external defibrillator training in London schools

**DOI:** 10.1186/cc14493

**Published:** 2015-03-16

**Authors:** J Salciccioli, D Marshall, M Sykes, A Wood, S Joppa, M Sinha, PB Lim

**Affiliations:** 1 Imperial College London, UK

## Introduction

Mortality from cardiac arrest remains high [1]. Bystander cardiopulmonary resuscitation (CPR) and the use of automated external defibrillators (AED) are two of the most important factors favouring survival [2]. CPR/AED training in schools is a recommended intervention for significantly improving training rates across a large population [3]. The current practice for CPR/AED training in London schools is unknown. The primary aim of this study was to assess current practices relating to CPR and AED training in London secondary schools.

## Methods

We conducted a registered audit of CPR and AED training in London schools. Secondary schools were identified via web links for each of the London Borough Councils. Telephone interviews with school staff familiar with CPR and AED training practices were conducted prospectively using a standardized web-based survey. All survey response data were captured electronically. We defined universal training as any programme which delivers CPR and AED training to all students in the school. We used simple descriptive statistics to summarise the results.

## Results

A total of 51 schools completed the survey covering an estimated student population of 54,037. There were four (8%) schools that provide universal training programmes and an additional 23 (45%) offer optional training programmes for students. There were 16 (31%) schools which have an AED available on the school premises. The most common reasons for not having a universal CPR training programme is the requirement for additional class time (15/51; 29%) and that funding is unavailable for such a programme (12/51; 24%). There were three students who died from sudden cardiac arrest over the period of the past 10 years.

## Conclusion

CPR and AED training rates in London secondary schools are low. The majority of schools do not have an AED available on premises. The most common reason for not providing CPR training is the requirement for additional class time. These data highlight an opportunity to vastly improve CPR training rates in a large population. Future studies should assess programmes which are cost-effective and which do not require significant amounts of additional class time.
